# Early detection of secondary damage in ipsilateral thalamus after acute infarction at unilateral corona radiata by diffusion tensor imaging and magnetic resonance spectroscopy

**DOI:** 10.1186/1471-2377-11-49

**Published:** 2011-05-05

**Authors:** Chuo Li, Xueying Ling, Sirun Liu, Anding Xu, Yusheng Zhang, Shihui Xing, Zhong Pei, Jinsheng Zeng

**Affiliations:** 1 Department of Neurology and Stroke Center, the First Affiliated Hospital, Sun Yat-Sen University, No 58, Zhongshan Road 2, Guangzhou, 510080, China; 2 Medical Imaging Centre, the First Affiliated Hospital, Jinan University, No.613, West Huangpu Road, Guangzhou, 510630, China; 3 Department of Neurology, the First Affiliated Hospital, Jinan University, No.613, West Huangpu Road, Guangzhou, 510630, China

## Abstract

**Background:**

Traditional magnetic resonance (MR) imaging can identify abnormal changes in ipsilateral thalamus in patients with unilateral middle cerebral artery (MCA) infarcts. However, it is difficult to demonstrate these early changes quantitatively. Diffusion tensor imaging (DTI) and proton magnetic resonance spectroscopy (MRS) are potentially sensitive and quantitative methods of detection in examining changes of tissue microstructure and metabolism. In this study, We used both DTI and MRS to examine possible secondary damage of thalamus in patients with corona radiata infarction.

**Methods:**

Twelve patients with unilateral corona radiata infarction underwent MR imaging including DTI and MRS at one week (W1), four weeks (W4), and twelve weeks (W12) after onset of stroke. Twelve age-matched controls were imaged. Mean diffusivity (MD), fractional anisotropy (FA), N-acetylaspartate (NAA), choline(Cho), and creatine(Cr) were measured in thalami.

**Results:**

T1-weighted fluid attenuation inversion recovery (FLAIR), T2-weighted, and T2-FLAIR imaging showed an infarct at unilateral corona radiate but no other lesion in each patient brain. In patients, MD was significantly increased at W12, compared to W1 and W4 (all *P*< 0.05). NAA was significantly decreased at W4 compared to W1, and at W12 compared to W4 (all *P*< 0.05) in the ipsilateral thalamus. There was no significant change in FA, Cho, or Cr in the ipsilateral thalamus from W1 to W12. Spearman's rank correlation analysis revealed a significant negative correlation between MD and the peak area of NAA, Cho, and Cr at W1, W4, and W12 and a significant positive correlation of FA with NAA at W1.

**Conclusions:**

These findings indicate that DTI and MRS can detect the early changes indicating secondary damage in the ipsilateral thalamus after unilateral corona radiata infarction. MRS may reveal the progressive course of damage in the ipsilateral thalamus over time.

## Background

Secondary damage in the ipsilateral thalamus separate from the primary infarcted area has been demonstrated by a number of studies [1-9]. In rats with distal middle cerebral artery occlusion (MCAO), secondary neuronal degeneration and gliosis in non-ischemic ipsilateral thalamus were observed at 1 week after ischemic injury of the thalamocortical pathway [5]. In addition, in patients with unilateral middle cerebral artery (MCA) infarcts, 47% showed hyperintensity within the ipsilateral thalamus 1-12 months after stroke on T2-weighted images. Post-mortem examination in one patient demonstrated neuronal loss and gliosis at this thalamic hyperintensity four months after stroke [6]. Although T2-weighted imaging shows abnormal changes in thalamus, it is still difficult to quantify the microstructural and metabolic changes. Moreover, in monkeys with MCAO, pathological analyses have shown a decrease in the number of neurons in the thalamus ipsilateral to the infarcted hemisphere, as early as 4 weeks. No signs of abnormalities were seen on T2-weighted images at that time [3], suggesting that traditional MR imaging could not demonstrate these secondary changes in the ipsilateral thalamus at this early stage.

Diffusion tensor imaging (DTI) is able to characterize brain tissue structure by measurement of water diffusion, and it can detect changes not identified by traditional MR imaging. It is expressed as two indices, mean diffusivity (MD) and fractional anisotropy (FA). MD is a parameter of magnitude of average molecular motion considered in all directions. FA is a quantitative measure of the degree of anisotropy, which is characterized by the random motion of water preferentially restricted to movement in one direction [10,11]. Studies in patients with MCA infarcts have demonstrated a significantly increased MD in the ipsilateral thalamus, reflecting less restriction in the movement of water molecules, which suggests the loss of thalamic components [12]. However, the underlying pathological changes in the thalamus are still uncertain.

Proton magnetic resonance spectroscopy (MRS) allows for non-invasive imaging of metabolic changes within the brain. Evidence of neuronal or axonal loss and damage can be obtained from measurement of N-acetylaspartate (NAA), which is considered a marker for neuronal and axonal integrity, viability, activity, and function [13-18]. Several studies have shown a reduction of NAA/creatine(Cr) values and NAA concentration in the thalami of patients with depression or epilepsy. No abnormalities are typically seen in these cases with traditional MR imaging, and these findings have been thought to represent neuronal and axonal degeneration and dysfunction [13,15-17]. MRS could be used to investigate neuronal and axonal density and function through NAA changes without the potential effect of microglial activation on diffusion, which can make DTI changes challenging to interpret. To observe secondary changes in the thalamus after stroke using DTI combined with MRS may reveal the pathological basis of the imaging abnormalities noted on DTI.

The corona radiata is the core of the hemispheric white matter, and its infarction may compromise the integrity of the corticospinal tract and thalamic radiations, causing sensation, movement, and cognitive dysfunction [19-22]. Hervé et al. revealed that secondary injuries in the ipsilateral thalamus were present in cases of MCA infarction with cortical involvement, shown by DTI between the first and sixth month after stroke onset [12]. It is still unknown whether similar findings are seen in cases of impairment of thalamocortical pathways due to an acute corona radiata infarction without cortical involvement. The goal of the present study is to detect early secondary damage in the ipsilateral thalamus in patients following acute unilateral infarction of the corona radiata using DTI and MRS, and to further explore possible associations between pathological changes and DTI abnormities in these patients.

## Methods

### Subjects

The research protocol was approved by the medical ethical committee of the First Affiliated Hospital, Sun Yat-Sen University for clinical research, and informed consent was signed from all participants. We selected 12 consecutive patients (7 male, 5 female) between October 2008 and December 2009 in Department of Neurology and Stroke Center, the First Affiliated Hospital, Sun Yat-Sen University, within 7 days of a focal infarct in the unilateral corona radiata, without any other signal abnormalities on T1-weighted fluid attenuation inversion recovery (FLAIR) and T2-weighted images. Mean age was 62.7 ± 6.5. Patients with unstable vital signs or a history of central nervous system disorders were excluded. Demographic characteristics and vascular risk factors were recorded in each patient. All patients were investigated with DTI and MRS at 1 week (6 ± 1 days, W1), 4 weeks (28 ± 3 days, W4), and 12 weeks (85 ± 4 days, W12) following a pre-defined protocol. Infarct volume was estimated from the T2-weighted FLAIR images at W12. Controls were 12 healthy age-matched volunteers recruited over the same period examined once by DTI and MRS.

### MRI Protocol

**Figure 1:**
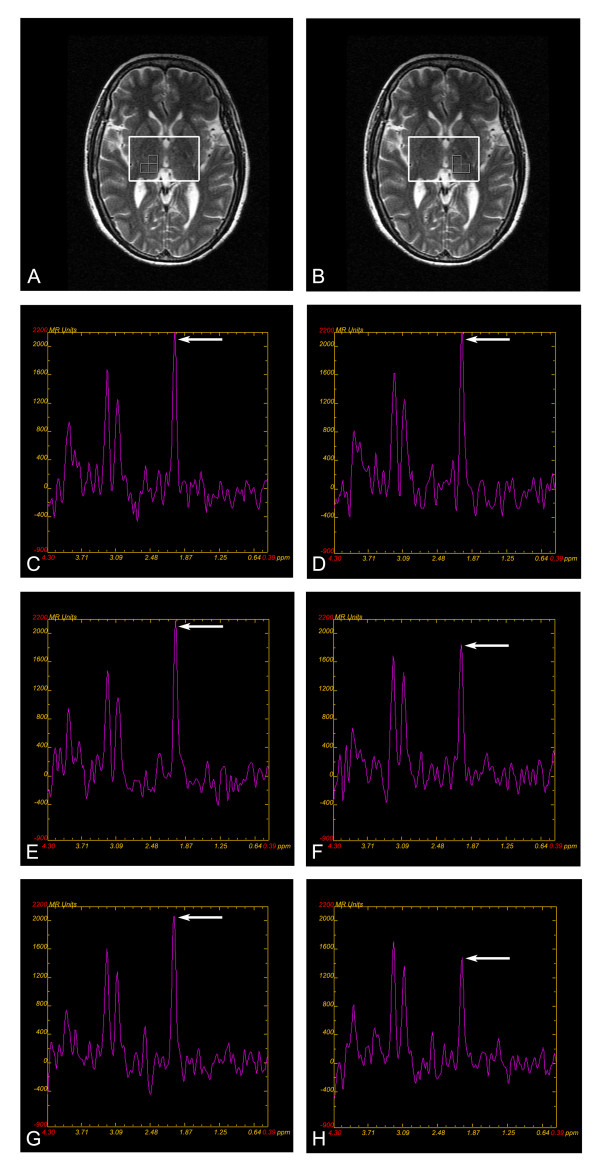
**MRS volume of interest in the right and left thalami and a sample of spectra from a patient**. (A) (B) Magnetic resonance spectroscopy (MRS) of the thalamus. T2-weighted image in the axial plane. Placement of the VOI covering the right and left thalami; multi-voxel spectroscopy technique; data is obtained from three adjacent voxels in each side thalamus. Mean peak areas of NAA, Cho and Cr values were determined by averaging three voxels in each side of the thalamus. (C)-(H) A sample of spectra from a patient depicting the values of analyzed metabolites. (C) (D) The spectrum of the ipsilateral and contralateral thalamus at W1. Arrows show NAA peak, which indicates approximately equal NAA value in ipsilateral and contralateral thalamus. (E) (F) The spectrum of the ipsilateral and contralateral thalamus at W4. Arrows show NAA peak, which indicates NAA value is lower in the in ipsilateral than that in contralateral thalamus. (G) (H) The spectrum of the ipsilateral and contralateral thalamus at W12. Arrows show NAA peak, which indicates NAA value is further lower in the in ipsilateral than that in contralateral thalamus

MRIs were performed using a 1.5-tesla MRI system (Signa General Electric Medical Systems, Milwaukee, Wis., USA) equipped with gradient hardware allowing up to 23 mT/m. T1-FLAIR was obtained within the first week. Fast spin echo T2-weighted imaging, T2-FLAIR, DTI, and proton MRS imaging were performed at W1, W4, and W12. Typical acquisition parameters of conventional MR imaging were: T1-FLAIR (6 mm thick, 2 mm gap, TR 1,475 ms/TE 21.3 ms/TI 750.0 ms), T2-FLAIR (3 mm thick, 0 mm gap, TR8,000/ TE120/TI2,200 ms). DTI was obtained with an echo planar imaging sequence (3 mm thick, 0 mm gap, TR 10,000/TE 100 ms, NEX = 1, matrix 128 × 128, field of view 24 × 24 cm). DTI was acquired with a *b *factor of 1,000 s/mm^2 ^and diffusion-sensitive gradients were applied along 15 gradient directions. In addition, a reference image without diffusion weighting (*b *= 0 s/mm^2 ^) was acquired. 15 repeats were acquired and averaged to improve signal to noise ratio. The scanning baseline was parallel to the antero-posterior commissural (ACPC) line. Axial slices covering the whole brain were obtained using a fast spin echo T2 (3 mm thick, 0 mm gap, TR 5,660 ms/TE 107.7 ms) serving as a matrix for the spectroscopic measurement and serving to position a spectroscopic volume of interest (VOI). Positioning of VOI was adjusted to the individual anatomy on T2 images covering the left and right thalamus (Figure 1). The proton spectroscopic data were acquired by using a chemical shift imaging method (2D PROBE-SI PRESS S 144TE) with these basic parameters: TR 1,000 ms/TE 144.0 ms, NEX = 1, 10 mm thick, voxel dimension 7.5 mm × 7.5 mm × 10 mm (0.5625 mL), field of view 24 × 24 cm. Before spectra acquisition, water suppression was performed to obtain an adequate visualization of the peaks of the metabolites. Vacuum fixation cushions were used to limit subject head movement.

### Image Post-processing

An experienced radiographer carried out image post-processing without knowing the purpose of the study. The DTI data were transferred to a workstation and spatially filtered using a median 3 × 3 filter. Acquiring the DTI data with the dual-echo EPI sequence and ramp sampling considerably reduced, but did not eliminate, the geometric distortions. We corrected the residual distortions by registering the diffusion weighted imaging with the reference image using the two-dimensional module of the automated image registration package (eight-parameter with perspective) [23]. With this correction, the DTI parameters were calculated on a pixel-by-pixel basis. Using ADW4.2 Software (Signa General Electric Medical Systems, Milwaukee, Wis., USA), regions of interest (ROIs) covering the entire thalamus on each side were manually delineated. According to the methods used by Hervé et. al., the medial boundaries of the thalamus were verified by the limits of cerebrospinal fluid ventricle on T2-FLAIR, and the lateral limits were defined by the internal capsule on FA maps [12]. DTI maps were calculated at W1 and on follow-up scans. The quantitative MD [derived from the trace of the diffusion tensor, MD = trace (D)/3] and FA data in thalamus obtained at different time points were analyzed.

The post-processing and quantification steps were automatically done using ADW4.2 Software. Spectral post-processing was comprised of line broadening, zero-filling, reducing the residual water resonance, linear baseline correction, and peak integration. Data were obtained from three adjacent voxels in the thalami on each side (Figure 1). Each spectrum was evaluated for the presence of NAA, choline (Cho), and Cr. The peak areas of NAA, Cho, and Cr were present in each spectrum for further evaluation.

### Statistical Analysis

MD, FA values, and proton metabolite levels obtained at W1, W4, and W12 in patients were first compared with those of controls with a two-tailed Student's *t *test. DTI parameters (MD and FA values) and metabolite levels (peak areas of NAA, Cho, and Cr) obtained at W1, W4, and W12 were analyzed using the Tukey test for multiple testing. To reduce inter-patient and inter-exam variability, asymmetry indices (ipsilateral/contralateral to the ischemic lesion) of MD, FA values, and peak areas of NAA, Cho, Cr obtained in patients, were calculated according to methods described by Hervé et al. [12], and further analyzed using Tukey test for multiple testing. Finally, the associations between MD, FA, and the peak area of NAA, Cho, and Cr were assessed with Spearman correlation analysis for the 12 patients at W1, W4, and W12. All analyses were performed using SPSS for Windows software, version 10.0, and the significance level was set at *P *< 0.05.

## Results

Twelve patients (7 male and 5 female) and 12 controls (7 male and 5 female) were recruited in the study. All patients and controls were right handed. Each patient had one or more vascular risk factors (Table 1).

**Table 1:** Patient demographics and clinical data

No.	Age (years)	Gender	Vascular factors	Infarct on T2-FLAIR images	
				
				Side	**Volume of lesion(mm**^**3**^**)**
1	59	M	Hypertension	L	11162
2	60	M	Hypertension, diabetes mellitus tobacco use	R	14497
3	67	F	Hypertension, Hypercholesterolemia, diabetes mellitus	L	13998
4	71	F	Diabetes mellitus	R	17018
5	66	M	Hypertension, tobacco use, overweight	L	13298
6	69	M	Hypertension	L	10966
7	55	F	Hypertension	R	12001
8	52	M	Hypertension, tobacco use	R	24893
9	70	M	Hypertension, tobacco use	L	24487
10	68	F	Hypertension	L	12104
11	58	M	Hypertension	R	17659
12	57	F	Diabetes mellitus	R	15300

F: female; M: male; L: left; R: right; FLAIR: fluid attenuated inversion recovery. The volume of the infarcted lesion was calculated on T2-FLAIR images at W12. Controls were recruited specifically to match age and gender of patients.

**Figure 2:**
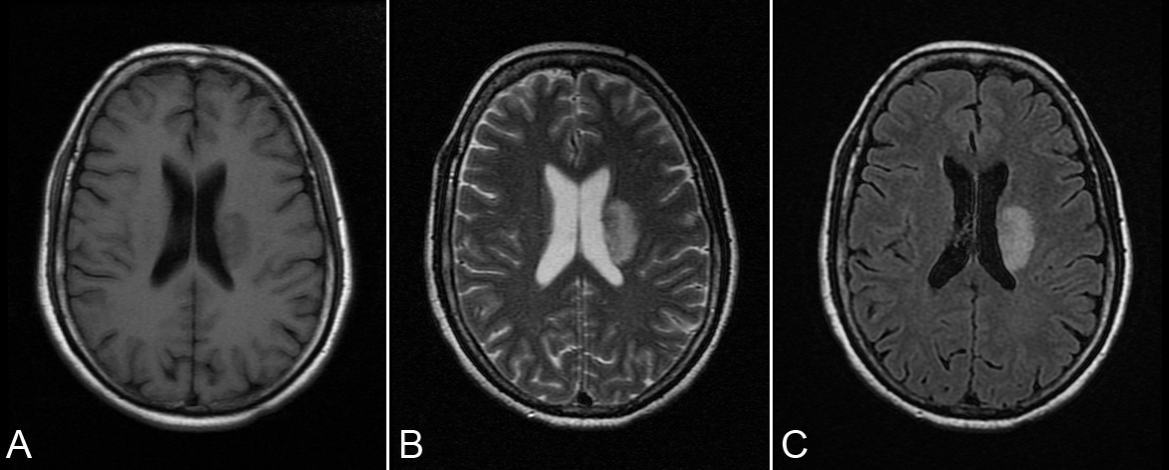
**T1-FLAIR, T2-weighted and T2-FLAIR images from a patient show focal infarct at left corona radiate**. (A) T1-FLAIR obtained 2 days after stroke show a hypointense area at left corona radiate. (B) T2-weighted image and (C) T2-FLAIR images obtained at W1 show a hyperintense area at left corona radiate.

Thirty-six examinations in patients and 12 examinations in controls were performed. Within the first week from stroke onset, T2-weighted and T2-FLAIR images showed focal hyperintensity confined to the unilateral corona radiata in each patient (Figure 2), and there was no abnormal signal outside the corona radiata. The infarct volume varied from 11162 to 24893 mm^3 ^(15615 ± 4740 mm^3^) on T2 images at W12. No definite abnormal signal was observed in the brains of control patients, nor in thalami on T1-FLAIR, T2-weighted and T2-FLAIR images throughout the duration of the study.

In controls, in the absence of any significant difference between the left and right thalami for MD (mean values: 0.833 ± 0.047 × 10^-3^mm^2^/s and 0.837 ± 0.047 × 10^-3^mm^2^/s, respectively, *P *= 0.382), FA (mean values: 0.345 ± 0.072 and 0.349 ± 0.053, *P *= 0.443), NAA (mean values: 13897 ± 2148 and 13946 ± 1693, respectively, *P *= 0.884), Cho (mean values: 9710 ± 1971 and 9502 ± 2331, respectively, *P *= 0.307) and Cr (mean values: 8030 ± 1464 and 7933 ± 1494, respectively, *P *= 0.318) the data obtained in the right thalamus were selected to compare absolute values in patients and controls.

On DTI, MD values at W1 in the ipsilateral and contralateral thalamus to the ischemic lesion in patients were significantly higher than those of controls (*P *= 0.010 and *P *= 0.025, respectively). There was a significant time effect on MD calculated in the ipsilateral, but not in the contralateral thalamus. A significant increase of MD in the ipsilateral thalamus was found between W1 and W12 and between W4 and W12 (*P *= 0.012 and *P *= 0.003 respectively). However, no obvious MD value changes were found in contralateral thalami (Table 2). There were no significant changes in FA in the ipsilateral and contralateral thalami from W1 to W12 (Table 2).

**Table 2:** MRI parameter values in thalamus in patients and controls (mean ± SD)

MRI Parameter	Ipsilateral Thalamus			Contralateral Thalamus			Right Thalamus in controls
		
	W1	W4	W12	W1	W4	W12	
MD(×10^-3^mm^2^/s)	0.877 ± 0.059^a^	0.863 ± 0.051^c^	0.954 ± 0.074^abcd^	0.866 ± 0.046^a^	0.872 ± 0.044^a^	0.869 ± 0.043^a^	0.837 ± 0.047
FA	0.332 ± 0.077	0.321 ± 0.063	0.315 ± 0.075	0.344 ± 0.046	0.352 ± 0.048	0.349 ± 0.058	0.349 ± 0.053
NAA	12911 ± 1592^a^	11139 ± 1943^abc^	9365 ± 1703^abcd^	13471 ± 1632	13220 ± 1830	13788 ± 1298	13946 ± 1693
Cho	8907 ± 1294	8893 ± 1963	8848 ± 1689	9762 ± 2397	9008 ± 1700	9343 ± 963	9502 ± 2331
Cr	7808 ± 1310	7585 ± 1539	7213 ± 1288	7995 ± 1317	7955 ± 1064	7751 ± 716	7933 ± 1494

ROI: region of interest; MD: mean diffusivity; FA: fractional anisotropy; NAA: N-acetylaspartate; Cho: choline; Cr: creatine; W1: the first week; W4: the fourth week; W12: the twelfth week.

^a^*P*< 0.05, Student's *t *test, compared to the control group; ^b^*P*< 0.05, Student's *t *test, compared to contralateral thalamus in patient group at the same time point; ^c^*P*< 0.05, Tukey test, compared to W1 in ipsilateral thalamus; ^d^*P*< 0.05, Tukey test, compared to W4 in ipsilateral thalamus.

Compared with control thalami, NAA obtained at W1 in ipsilateral thalami in patients showed a significant difference (*P *= 0.048). There was a significant time effect on MRS metabolites measured on the ipsilateral side. NAA in ipsilateral thalami were significantly decreased between W1 and W4, between W1 and W12, and between W4 and W12 (*P *= 0.048, *P *= 0.000 and *P *= 0.047), however, no obvious NAA changes were found in the contralateral thalami (Table 2) (Figure 1). There were no significant changes in Cho and Cr measured in the ipsilateral or the contralateral thalami from W1 to W12 (Table 2).

**Figure 3:**
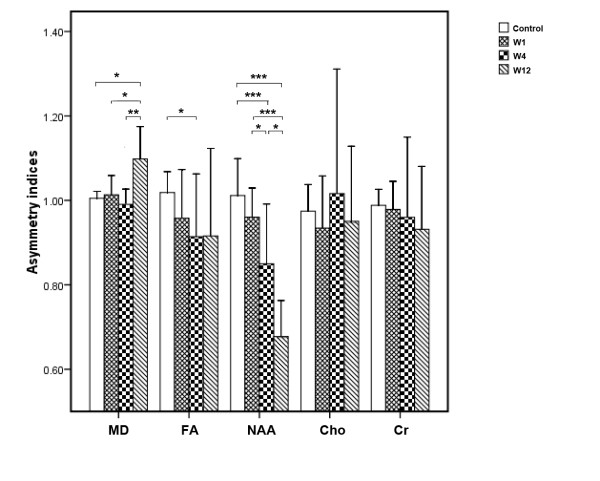
**Thalamic asymmetry indices of MD, FA and of peak areas of NAA, Cho and Cr values**. MD: mean diffusivity; FA: fractional anisotropy; NAA: N-acetylaspartate; Cho: choline; Cr: creatine; W1: the first week; W4: the fourth week; W12: the twelfth week. Asymmetry indices: thalamic ipsilateral/contralateral MRI Parameter value in patients and right/left MRI parameter value in controls. *P< 0.05, **P< 0.01, ***P< 0.001.

Contrasting data from patients, the asymmetry indices for MD were markedly increased at W12, compared with those at W4 and W1, and with controls. The asymmetry indices for FA were significantly decreased at W4, compared with those of controls. The asymmetry indices for peak areas of NAA were significantly decreased between W1 and W4, between W1 and W12, and between W4 and W12 (*P *= 0.034, *P *= 0.000 and *P *= 0.001). There was no significant changes in the asymmetry indices for peak areas of Cho or Cr from W1 to W12 (Table 3) (Figure 3).

**Table 3:** Asymmetry indices of MRI Parameter value

	Patients			Controls
		
	W1	W4	W12	
MD	1.013 ± 0.046	0.991 ± 0.036	1.098 ± 0.076^abc^	1.005 ± 0.016
FA	0.958 ± 0.115	0.914 ± 0.148^c^	0.915 ± 0.207	1.019 ± 0.049
NAA	0.961 ± 0.069	0.850 ± 0.142^ac^	0.678 ± 0.085^abc^	1.012 ± 0.087
Cho	0.935 ± 0.123	1.016 ± 0.295	0.951 ± 0.178	0.975 ± 0.064
Cr	0.979 ± 0.067	0.960 ± 0.190	0.932 ± 0.149	0.988 ± 0.038

MD: mean diffusivity; FA: fractional anisotropy; NAA: N-acetylaspartate; Cho: choline; Cr: creatine; W1: the first week; W4: the fourth week; W12: the twelfth week.

Asymmetry indices: thalamic ipsilateral/contralateral MRI Parameter value in patients and right/left MRI Parameter value in controls.

^a^*P*< 0.05, Tukey test, compared to W1; ^b^*P*< 0.05, Tukey test, compared to W4; ^c^*P*< 0.05, Student's *t *test, compared to the control group.

Spearman's correlation (r_s_) between DTI parameters (MD, FA) with the corresponding MRS parameters (NAA, Cho) in patients showed a significant negative correlation of MD with NAA, Cho, and Cr at W1 (MD with NAA: r_s _= -0.816, *P *= 0.001; MD with Cho: r_s _= -0.872, *P*< 0.001; MD with Cr: r_s _= -0.651, *P *= 0.022 ), W4 (MD with NAA: r_s _= -0.797, *P *= 0.002; MD with Cho: r_s _= -0.622, *P *= 0.031; MD with Cr: r_s _= -0.692, *P *= 0.013), and W12 (MD with NAA: r_s _= -0.722, *P *= 0.008; MD with Cho: r_s _= -0.823, *P *= 0.001; MD with Cr: r_s _= -0.767, *P *= 0.004), and a significant positive correlation of FA with NAA at W1 (r_s _= .0.578, *P *= 0.049). There was no significant correlation between FA and NAA at W4 and W12, between FA and Cho at W1, W4, and W12.

## Discussion

To the best of our knowledge, this is the first prospective study using DTI and MRS to observe secondary changes in the thalamus, remote from a single subcortical cerebral infarction within the territory of the MCA. A significantly increased MD at W12 and a significantly decreased NAA at W4 and W12 were observed in the thalamus ipsilateral to the infarction. This suggests that both DTI and MRS detect certain aspects of early secondary thalamic damage in patients with acute infarction of the corona radiata, and MRS might be more sensitive than DTI in detecting secondary thalamic damage in the early stages of a stroke. Moreover, a significant negative correlation between MD and NAA at W1, W4, and W12 in the ipsilateral thalamus was found, suggesting that the structural loss in neurons is consistent with the dysfunction.

In this study, asymmetry indices comparing MD, FA, and peak areas of NAA, Cho, and Cr in thalamus were evaluated to reduce inter-patient and inter-exam variability. Analysis of MD, FA, and peak areas of NAA, Cho, and Cr, together with asymmetry indices of those parameters, showed that NAA was significantly decreased at W4 and W12, while MD was increased at W12 compared to W1 and controls. However, there were no significant differences in MD and NAA at W1 compared to controls by analysis of asymmetry indices. DTI and MRS parameter values in the contralateral thalamus were stable during the observation period, and thus can be regarded as an internal reference for calculating asymmetry indices. By using this method, effects of hypertension or other factors on MRI data may be ruled out; furthermore, the consistency of trends in MD and NAA changes shown by asymmetry indices and absolute values suggests that asymmetry indices might be a more reliable and reasonable method.

Increased MD in the ipsilateral thalamus may be a reflection of secondary degeneration due to disruption of white matter tracts linking the thalamus to other structures, specifically degeneration of cortico-thalamic or thalamocortical pathways [12,24]. A significant increase of MD in the ipsilateral thalamus was found between W1 and W12, and between W4 and W12, but there were no significant changes between W1 and W4. Such increases in MD in the thalamus have also been described in patients with cerebral autosomal dominant arteriopathy with subcortical infarcts and leukoencephalopathy (CADASIL) [24,25]. Similarly, in a study on patients with MCA infarcts, an increase in MD was observed between the first and the sixth month in the ipsilateral thalamus in patients with cerebral cortex involvement [12]. The difference in our study is that the infarction was located in the corona radiata, without cortical involvement, thus excluding the effects of cortical damage on the thalamus. The abnormal diffusion observed in the thalamus in our study suggest that the secondary damage is cumulative from W1 to W12, and it is more obvious at later stages after infarction. FA is a measure of the directionality of diffusion, and is greater in white matter tracts than in grey matter. In our study, there was no significant FA difference observed in thalami between patients and controls, between thalami ipsilateral and contralateral to the infarction, which may be due to fewer oriented fiber bundles, and more nuclei in parallel within the thalamus. It was noteworthy that, in a study on CADASIL, significant reductions in anisotropy were found in thalami on both sides in patients compared to the controls [24]. It is probable that longer follow-up and more sophisticated image analysis techniques are required for determining FA changes in the thalamus ipsilateral to a remote infarction. Accordingly, these results provide evidence that DTI may detect early damage to the thalamus ipsilateral to the infarction, and increased MD in the ipsilateral thalamus might have diagnostic value in demonstrating and evaluating secondary damage, although it may not show the changes of dynamic process within 4 weeks after stroke onset.

Animal and autopsy studies have shown that neuronal degeneration and a reduction in the number of nerve cells occur in the ipsilateral thalamus after MCA infarction [3-6,8]. Our analysis of spectroscopic images acquired with proton MRS show a significantly progressive decrease in the ipsilateral thalamic NAA in patients from W1 to W4, and W4 to W12, indicating neuronal and/or axonal loss and dysfunction. Such changes in thalamus are in line with previously-reported findings in the ipsilateral thalamus after infarction [3-6,8]. It is presumable that after corona radiata infarction, thalamic neuronal anterograde and retrograde degeneration induces neuronal and/or axonal loss and dysfunction in the ipsilateral thalamus, which results in damage to cortico-thalamic or thalamocortical fibers. Cho/Cr increases in the thalami of depressive patients have been associated with membrane phospholipids related to metabolism abnormality [17]. In our study, there were no significant changes of Cho during the observation period. This suggests that the pathological alterations in membrane turnover in the thalamus with secondary damage might be a chronic process. Moreover, NAA decreased obviously within 4 weeks, but no significant changes in MD were found during this stage. These results indicate that MRS can also detect early damage in the ipsilateral thalamus after infarction MRS is more sensitive in revealing the dynamic process of secondary damage in the ipsilateral thalamus in the early stages of stroke.

Correlations between DTI and MRS parameters have been reported in healthy volunteers and primary progressive multiple sclerosis (ppMS) patients. A negative correlation between NAA and ADC was found in ppMS patients and controls [26,27]. In our study, we also found a significant negative correlation between MD and NAA at W1, W4, and W12, suggesting that neuronal structure damage indicated by increased MD was approximately coincident with neuronal metabolism damage indicated by decreased NAA. This consistency of neuronal structure loss and dysfunction suggests a phenomenon of neuronal degeneration in the ipsilateral thalamus. In addition, FA correlates positively with NAA at W1, but showed no correlations at W4 and W12, suggesting that FA cannot reflect secondary damage process indicated by increased MD and decreased NAA in the ipsilateral thalamus. Cho showed negative correlations with MD, suggesting a constructive role of Cho in the preservation of neuron structures [26]. It is in agreement with a previous study on healthy controls in which a negative correlation of MD with Cho was shown [27]. Moreover, MD showed negative correlations to Cr suggests integrity of structure indicated by MD was associated with neuronal energy use and storage indicated by Cr in thalamus [28].

## Conclusion

Using DTI and MRS, we found significantly increased MD at W12, and decreased NAA at W4 and W12 in the ipsilateral thalamus in patients with acute unilateral infarction of the corona radiata, indicating notable secondary damage in the ispilateral thalamus in the early stages after stroke. DTI and MRS can detect early secondary damage in the ipsilateral thalamus, and MRS may reveal the dynamic process of damage in the ipsilateral thalamus. The pathologic basis behind the secondary damage in ipsilateral thalamus may be mainly a result of neuronal and/or axonal degeneration, loss, and dysfunction. Moreover, neuronal and/or axonal structural loss was consistent with the neuronal and/or axonal dysfunction.

## Competing interests

The authors declare that they have no competing interests.

## Authors' contributions

CL participated in the design of the study, collected the data and wrote the primary manuscript, XL carried out image postprocessing, SL conducted the MR images analysis, AX assisted with the data collection, YZ assisted with the statistical analysis, SX assisted with the data collection, PZ participated in the design of the study, JZ designed the study, interpreted the results and critically revised the manuscript, all authors read and approved the final manuscript.

## Pre-publication history

The pre-publication history for this paper can be accessed here:

http://www.biomedcentral.com/1471-2377/11/49/prepub
